# Nrf2 Activation Attenuates Acrylamide-Induced Neuropathy in Mice †

**DOI:** 10.3390/ijms22115995

**Published:** 2021-06-01

**Authors:** Chand Basha Davuljigari, Frederick Adams Ekuban, Cai Zong, Alzahraa A. M. Fergany, Kota Morikawa, Gaku Ichihara

**Affiliations:** 1 Department of Occupational and Environmental Health, Tokyo University of Science, Noda 278-8510, Japan; drchandbasha2012@gmail.com (C.B.D.); 3B18701@alumni.tus.ac.jp (F.A.E.); zongcai@rs.tus.ac.jp (C.Z.); Zhraa.fergany@alexu.edu.eg (A.A.M.F.); mkouta9331@gmail.com (K.M.); 2 Genetics and Genetic Engineering in Department of Animal Husbandry and Animal Wealth Development, Faculty of Veterinary Medicine, Alexandria University, Alexandria 21500, Egypt

**Keywords:** sulforaphane, Nrf2, acrylamide, neurotoxicity, noradrenergic axons, oxidative stress

## Abstract

Acrylamide is a well characterized neurotoxicant known to cause neuropathy and encephalopathy in humans and experimental animals. To investigate the role of nuclear factor erythroid 2-related factor 2 (Nrf2) in acrylamide-induced neuropathy, male C57Bl/6JJcl adult mice were exposed to acrylamide at 0, 200 or 300 ppm in drinking water and co-administered with subcutaneous injections of sulforaphane, a known activator of the Nrf2 signaling pathway at 0 or 25 mg/kg body weight daily for 4 weeks. Assessments for neurotoxicity, hepatotoxicity, oxidative stress as well as messenger RNA-expression analysis for Nrf2-antioxidant and pro-inflammatory cytokine genes were conducted. Relative to mice exposed only to acrylamide, co-administration of sulforaphane protected against acrylamide-induced neurotoxic effects such as increase in landing foot spread or decrease in density of noradrenergic axons as well as hepatic necrosis and hemorrhage. Moreover, co-administration of sulforaphane enhanced acrylamide-induced mRNA upregulation of Nrf2 and its downstream antioxidant proteins and suppressed acrylamide-induced mRNA upregulation of tumor necrosis factor alpha (TNF-α) and inducible nitric oxide synthase (iNOS) in the cerebral cortex. The results demonstrate that activation of the Nrf2 signaling pathway by co-treatment of sulforaphane provides protection against acrylamide-induced neurotoxicity through suppression of oxidative stress and inflammation. Nrf2 remains an important target for the strategic prevention of acrylamide-induced neurotoxicity.

## 1. Introduction

Acrylamide is a neurotoxicant widely used in many industrial applications [1]. As an electrophile, acrylamide is known to form reactive Michael-type adducts with nucleophilic residues in living organisms [2] and has been confirmed to exhibit neurotoxicity in both humans and experimental animals [3,4,5,6,7], as well as genotoxicity, reproductive toxicity and carcinogenicity in experimental animals [8,9,10,11,12]. Exposure to acrylamide is known to cause ascending central or peripheral axonopathies in humans and animals, thereby leading to sensory, motor, and autonomic dysfunctions [13].

The acrylamide-induced toxicities have been linked to the monomer form, which is mostly used for the synthesis of polyacrylamides, used in several applications, such as water purification, preparation of anti-grouting agents, among others [13]. Human exposure to acrylamide occurs through several anthropogenic means, such as tobacco smoke [14,15], occupational exposure and water pollution. In April 2002, researchers at the Swedish National Food Administration and the University of Stockholm, Sweden, reported the discovery of significant levels of acrylamide in various foods, such as coffee and potato crisps that have been thermally processed through a process known as the Maillard reaction [16,17]. Acrylamide is unintentionally generated when foods containing amino acids, such as asparagines, and carbohydrates containing reducing sugars, such as glucose, are processed under extremely hot conditions [17]. The levels of acrylamide generated are thus dependent on the cooking temperature, cooking time, water and moisture contents and the amounts of amino acid (asparagine) and reducing sugar (glucose) present in raw food [18,19,20]. Based on the wide range of food items that may contain acrylamide, avoidance of such foods or undercooking food for fear of generating acrylamide, could constitute health problems related to an unbalanced diet or possible microbiological infestation, respectively [21]. Thus, it seems that exposure of humans to acrylamide is inevitable, which could pose a great threat to human health and safety. Therefore, it is crucial to explore possible strategies that can offer protection against acrylamide-induced neurotoxic effects.

A large number of existing studies in the broader literature have established Nrf2 as the master regulator of cellular redox homeostasis [22,23,24]. Under conditions of oxidative or electrophilic stress, Nrf2 is detached from the stress sensor protein Kelch-like ECH-associated protein 1 (Keap1), and translocated to the nucleus, where it is heterodimerized with the small musculoaponeurotic fibrosarcoma (sMaf) protein [25,26,27,28,29]. The Nrf2-sMAF heterodimer then binds to the antioxidant or electrophile response element (ARE/EpRE), leading to the induction and transcription of several antioxidant and cytoprotective genes, including glutathione and thioredoxin systems [24,30], as well as several phases I, II and III drug-metabolizing enzymes involved in the regulation of oxidative stress [22,31,32,33]. 

Sulforaphane is a dietary phytochemical and an isothiocyanate abundantly present in cruciferous vegetables (e.g., broccoli, cabbage and Brussels sprouts), [34] that has been identified as a potent inducer of various cytoprotective metabolizing genes and enzymes through activation of the Nrf2 signaling pathway. Sulforaphane has been shown to offer protection against electrophiles, carcinogens, oxidative stress as well as inflammation [35,36,37]. 

A body of literature has reported that deletion of the *Nrf2* gene results in increased sensitivity to environmental electrophiles such as 1-bromopropane, cadmium, methyl mercury and 1,2-naphtaquenone among others [38,39,40,41,42]. Moreover, results from our recent publication have showed that deletion of the *Nrf2* gene increased the susceptibility of mice to acrylamide-induced neurotoxicity [43]. Notwithstanding these studies, it remains unknown whether activation of the Nrf2 signaling pathway offers protection against acrylamide-induced neurotoxicity in vivo. The aim of the present study was to determine whether the activation of the Nrf2 signaling pathway by sulforaphane offers protection against acrylamide-induced neurotoxicity and the related mechanisms of protection in mice.

## 2. Results

### 2.1. Changes in Body Weight 

Analysis of variance (ANOVA) followed by Dunnett’s multiple comparison showed that acrylamide dose-dependently and significantly decreased body weight at 300 ppm in mice groups untreated with sulforaphane but had no effect in the sulforaphane-treated mice groups (Figure S2; Table 1). Linear regression analysis showed a significant positive trend with acrylamide exposure level for body weight among the sulforaphane-untreated mice, in contrast to the sulforaphane co-treated mice, which did not show any significant change (Table 1). Multiple regression analysis showed no interaction for body weight indicating that sulforaphane does not change the effect of acrylamide dose level. Moreover, one model of multiple regression analysis free of interactions showed a significant effect for acrylamide exposure level and sulforaphane (Table 1).

### 2.2. Changes in Function

#### Landing Foot Spread and Hindlimb Clasping Effect Observation

ANOVA followed by Dunnett’s multiple comparison showed that acrylamide significantly increased the hindlimb splay at 200 and 300 ppm in both the sulforaphane-untreated and -treated mice. However, the extent of increase in the hindlimb splay in the sulforaphane-untreated mice was markedly higher relative to the sulforaphane-treated mice (Figure S3; Table 1). Multiple regression analysis did not show a significant interaction between acrylamide dose level and sulforaphane. Moreover, multiple regression analysis without interaction showed a significant effect for acrylamide exposure level but no significance for the effect for sulforaphane (Table 1).

**Figure 1:**
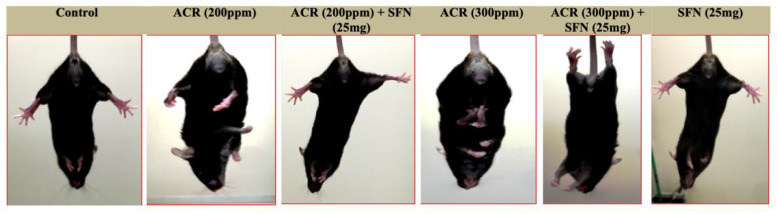
Representative images of mice showing various degrees of hindlimb clasping effect following exposure to acrylamide and sulforaphane for 4 weeks.

A qualitative-based observational assessment for motor dysfunction showed that acrylamide in sulforaphane-untreated mice induced an increased hindlimb clasping effect upon tail suspension relative to sulforaphane-treated mice, which showed reduced clasping effect and improved extension of the hindlimbs (Figure 1).

### 2.3. Changes in Monoaminergic Axons

#### Noradrenaline Transporter (NAT)-Immunoreactive (Noradrenergic) Axons

**Figure 2:**
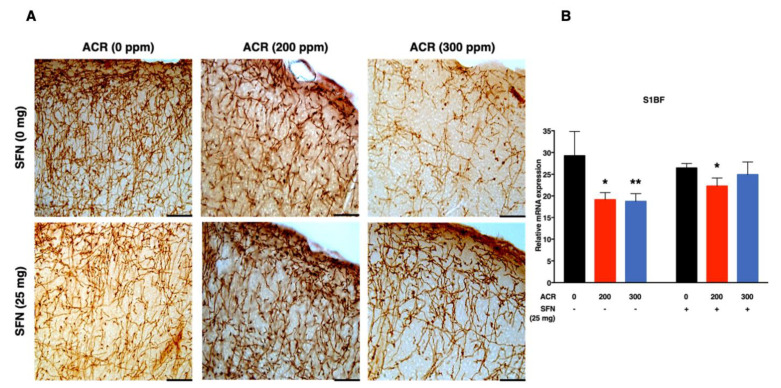
Representative photomicrographs (**A**) and density (**B**) of noradrenaline transporter (NAT)-immunoreactive axons in the Barrel field primary somatosensory cortex (S1BF) of mice following exposure to acrylamide and treatment with sulforaphane. Data are mean ± SD. * *p* < 0.05, ** *p* < 0.01, compared to the corresponding control (by ANOVA followed by Dunnett’s multiple comparison). Scale bars = 40 µm; *n* = 4.

**Figure 3:**
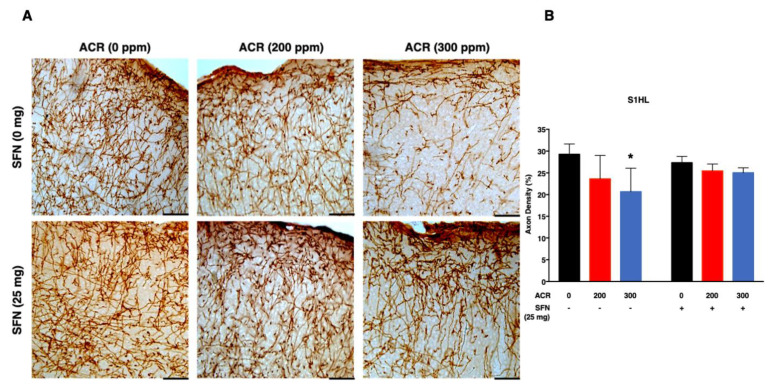
Representative photomicrographs (**A**) and density (**B**) of noradrenaline transporter (NAT)-immunoreactive axons in the hindlimb primary somatosensory cortex (S1HL) of mice following exposure to acrylamide and treatment with sulforaphane. Data are mean ± SD. * *p* < 0.05, compared to the corresponding control (by ANOVA followed by Dunnett’s multiple comparison) Scale bars = 40 µm; *n* = 4.

**Figure 4:**
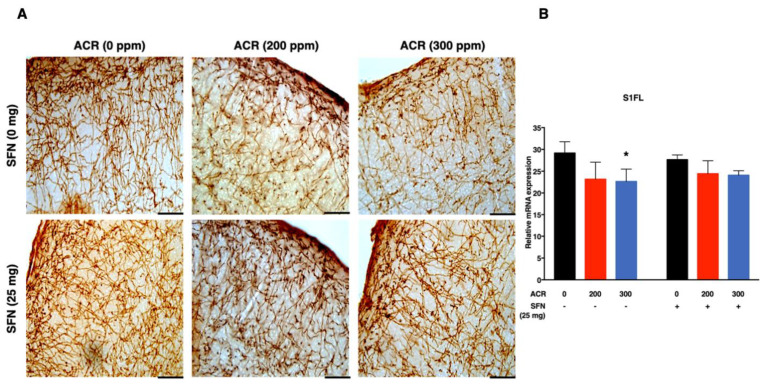
Representative photomicrographs (**A**) and density (**B**) of noradrenaline transporter (NAT)-immunoreactive axons in the forelimb primary somatosensory cortex (S1FL) of mice following exposure to acrylamide and treatment with sulforaphane. Data are mean ± SD. * *p* < 0.05, compared to the corresponding control (by ANOVA followed by Dunnett’s multiple comparison). Scale bars = 40 µm; *n* = 4.

**Figure 5:**
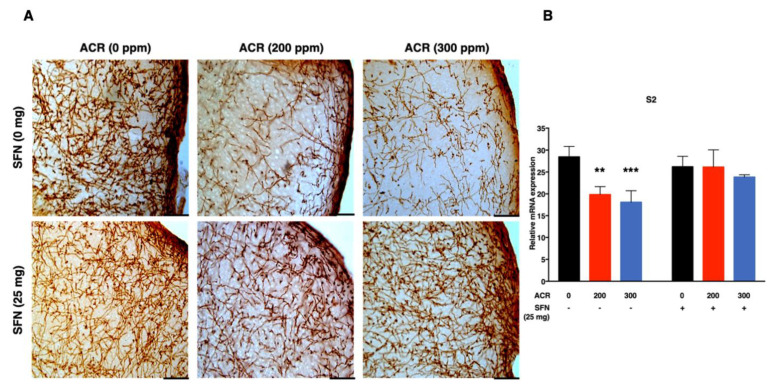
Representative photomicrographs (**A**) and density (**B**) of noradrenaline transporter (NAT)-immunoreactive axons in the secondary somatosensory cortex (S2) of mice following exposure to acrylamide and treatment with sulforaphane. Data are mean ± SD. ** *p* < 0.01, *** *p* < 0.001, compared to the corresponding control (by ANOVA followed by Dunnett’s multiple comparison). Scale bars = 40 µm; *n* = 4.

The density of noradrenergic-immunoreactive axons was quantified in the primary (S1HL, S1BF, S1FL) and secondary (S2) regions of the somatosensory cortex (Figure 2, Figure 3, Figure 4 and Figure 5). ANOVA followed by Dunnett’s multiple comparison showed that exposure to acrylamide significantly and dose-dependently decreased the density of noradrenergic axons in the S1HL and S1FL regions at 200 ppm as well as in the S1BF and S2 regions at 200 and 300 ppm in the sulforaphane-untreated mice. However, the same analysis showed that 200 and 300 ppm acrylamide significantly decreased the density of noradrenergic axons in the S1BF and S1FL regions of sulforaphane-treated mice, respectively. It is noteworthy that the decrease in density of noradrenergic axons was greater in the sulforaphane-untreated mice compared with the sulforaphane-treated mice (Figure 2, Figure 3, Figure 4 and Figure 5; Table S1). Moreover, it was noteworthy that the S2 region of the somatosensory cortex showed much higher sensitivity to acrylamide-induced degeneration of noradrenergic axons (Figure 5).

Simple regression analysis showed a significant positive trend with acrylamide exposure level in the S1HL, S1BF, S1FL and S2 regions of the somatosensory cortex in the sulforaphane-untreated mice as well as in the S1HL and S1FL regions of the sulforaphane-treated mice (Table 2). Multiple regression analysis showed a significant interaction between acrylamide exposure level and sulforaphane treatment for noradrenergic axons in the S1BF and S2 somatosensory cortex regions, indicating that the effect of acrylamide depends on the respective treatment of sulforaphane. A model of multiple regression analysis free of interaction showed a significant effect for acrylamide exposure level with no significant effect for sulforaphane in the S1HL and S1FL regions (Table 2).

### 2.4. Changes in mRNA Expression

#### 2.4.1. Nrf2-Antioxidant Genes

**Figure 6:**
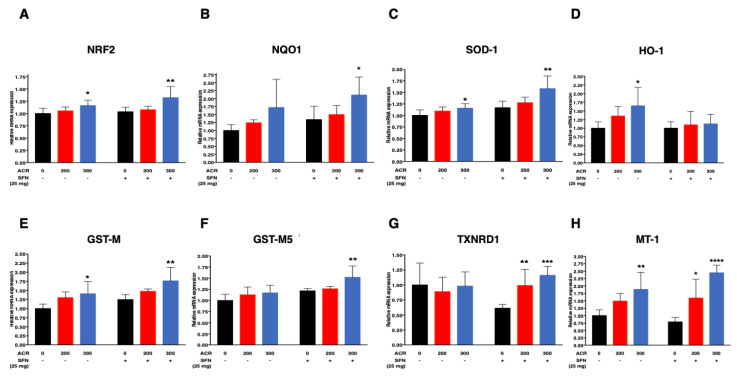
Effects of the combination of acrylamide and sulforaphane on relative mRNA expression of Nrf2 antioxidant genes in the cerebral cortex, Nrf2 (**A**), NQO1 (**B**), SOD-1 (**C**), HO-1 (**D**) GST-M (**E**), GST-M5 (**F**), TXNRD1 (**G**) and MT-1 (**H**) after exposure to acrylamide for 4 weeks. Data are mean ± SD. * *p* < 0.05, ** *p* < 0.01, *** *p* < 0.001, **** *p* < 0.0001, compared to the corresponding control (by ANOVA followed by Dunnett’s multiple comparison). [*n* = 6].

ANOVA followed by Dunnett’s multiple comparison showed that acrylamide at 300 ppm significantly induced the mRNA expression of Superoxide dismutase 1 (SOD-1), Heme oxygenase 1 (HO-1), Glutathione S transferase mu (GST-M), Nrf2 and metallothionein 1 (MT-1) in the sulforaphane-untreated mice. In contrast, sulforaphane induced a dose-dependent increase in the mRNA expression of SOD-1, NAD(P)H: quinone oxidoreductase 1(NQO1), Glutathione S transferase mu5 (GST-M5), GST-M and Nrf2 with marked and significant changes at 300 ppm (Figure 6A–C,E,F; Table S2). Furthermore, sulforaphane was associated with upregulation of Thioredoxin Reductase 1 (TXNRD1) and MT-1 in a dose-dependent manner, with marked and significant changes at 200 and 300 ppm acrylamide (Figure 6G,H; Table S2). Interestingly, acrylamide at 200 ppm did not induce significant upregulation of Nrf2-antioxidant gene expression both in sulforaphane-untreated and -treated mice. Furthermore, sulforaphane downregulated HO-1 mRNA expression (Figure 6D; Table S2).

Simple regression analysis showed a positive trend with the dose of acrylamide for SOD-1, NQO1, GST-M, Nrf2, and MT-1 in both treatment groups, and a significant increase in HO-1 in sulforaphane-untreated mice and in GST-M5 and TXNRD1 in sulforaphane-treated mice (Table 3). Multiple regression analysis showed significant interaction of acrylamide dose level with sulforaphane for TXNRD1 and MT-1. Other non-interaction models of multiple regression analysis showed that acrylamide exposure level correlated with increased mRNA expression of NQO1, HO-1, GST-M, and that sulforaphane significantly increased SOD-1, HO-1, GST-M5 and GST-M mRNA expression levels (Table 3).

#### 2.4.2. Pro-Inflammatory Cytokines

**Figure 7:**
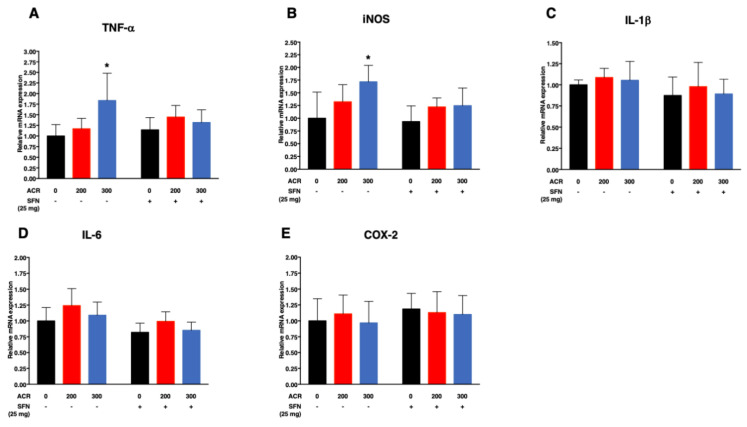
Effects of exposure to acrylamide and treatment with sulforaphane on relative mRNA expression of pro-inflammatory cytokines in the cerebral cortex; TNF-α (**A**), iNOS (**B**), IL-1β (**C**), IL-6 (**D**) and COX-2 (**E**) after exposure to acrylamide for 4 weeks. Data are mean ± SD. * *p* < 0.05, compared to the corresponding treatment control (by analysis of variance (ANOVA) followed by Dunnett’s multiple comparison), (*n* = 6).

ANOVA followed by Dunnett’s multiple comparison showed that exposure to acrylamide at 300 ppm significantly increased the mRNA expression levels of TNF-α and iNOS in the sulforaphane-untreated mice while sulforaphane abrogated such effects (Figure 7A,B; Table S3).

Simple regression analysis showed significant positive trend with the dose of acrylamide for TNF-α and iNOS in the sulforaphane-untreated mice but not in the sulforaphane-treated mice (Table 4). Multiple regression analysis showed no significant interaction of acrylamide exposure level and sulforaphane treatment for all the genes examined (TNF-α, iNOS, IL-1β, interleukin 1 beta; IL-6, interleukin 6 and COX-2, cyclooxygenase-2). Moreover, the same analysis model limited to the data of no-interaction showed significant positive effect for acrylamide exposure level on the mRNA expression TNF-α and iNOS as well as significant positive effect for sulforaphane on IL-6 (Table 4).

### 2.5. Changes in Glutathione and Malondialdehyde (MDA) Levels

ANOVA followed by Dunnett’s multiple comparison showed that sulforaphane significantly reduced malondialdehyde (MDA) levels when administered with 300 ppm acrylamide (Table 5; Figure S4). 

Single regression analysis showed significant positive trend with the dose of acrylamide for MDA among the sulforaphane groups. There was no significant effect for acrylamide exposure level or sulforaphane treatment on total glutathione, glutathione disulfide and glutathione-redox ratio (GSSG/GSH ratio) (Table 5). Multiple regression analysis showed significant interaction between acrylamide and sulforaphane for MDA, indicating different magnitude of effects by acrylamide depending on the treatment of sulforaphane. Moreover, multiple regression analysis without interaction showed no significant effect for acrylamide exposure level on total glutathione, GSSG and GSSG/GSH ratio but a significant effect for sulforaphane treatment on total glutathione levels (Table 5).

### 2.6. Effects of Acrylamide on Liver Histopathology

Histopathological examination of hematoxylin and eosin (H&E)-stained liver tissue samples showed normal liver morphology in the control (acrylamide 0 ppm) and sulforaphane-alone (25 mg/kg bw) mice (Figure S5). On the other hand, treatment with acrylamide up to 300 ppm produced extensive necrosis and severe hemorrhage (Figure S5). However, these pathological lesions were greatly attenuated in mice treated with sulforaphane. Mice of the latter group showed moderate to minimal hemorrhage with clearance of necrotic lesions (Figure S5).

## 3. Discussion

The present study investigated the role of Nrf2 in the process of acrylamide-induced neuropathy in mice upon activation by systemic administration of sulforaphane. The study used specific markers of neurotoxicity, including sensorimotor dysfunction and degeneration of monoaminergic-immunoreactive axons which were confirmed by landing foot spread test and immunohistochemistry respectively, and showed that activation of the Nrf2-signaling pathway by co-administration of sulforaphane offers protection against acrylamide-induced neurotoxicity in mice. Recent work by our laboratory using a gene deletion model of Nrf2-knockout mice have demonstrated the pivotal role of the Nrf2-dependent transcription offering protection against acrylamide-induced neurotoxicity such as sensorimotor dysfunction, degeneration of monoaminergic axons associated with activation of microglia [43]. Moreover, deletion of Nrf2 was shown to enhance acrylamide-induced pro-inflammatory cytokine genes and suppression of Nrf2-antioxidant genes, thereby establishing the importance of the Nrf2 gene [43].

Sulforaphane is a well-recognized inducer of the Nrf2-ARE signaling pathway which ultimately results in the induction of several phase II and III cytoprotective genes and enzymes that provide protection against various degrees of electrophilic attack caused by xenobiotics [36,44]. As such, sulforaphane has been shown to have neuroprotective potential in various neurological disorders [45,46,47,48]. 

A key mechanism on how sulforaphane activates the Nrf2 signaling pathway has been associated with covalent modification of Keap1. As an electrophile, sulforaphane activates the Nrf2-ARE signaling pathway by reacting with sulfhydryl or thiol functional groups of cysteine residues found in Keap1, particularly Cys-151, which results in the termination of the Culin-3 ubiquitin ligase-mediated proteosomal degradation and dissociation and subsequent build-up of Nrf2 expression [49,50,51].

The neuroprotective role of sulforaphane has already been reported in several experimental models. For example, pre-treatment with sulforaphane effectively ameliorated **6**-hydroxydopamine (6-OHDA) and hydrogen peroxide (H_2_O_2_)-induced oxidative stress and cytotoxicity in dopaminergic neuroblastoma SH-SY5Y cell line [47]. Moreover, sulforaphane provided protection against cell damage induced by rotenone in PC12 cells [52] as well as inhibition of rotenone-induced deficiency of locomotor activity and loss of dopaminergic neurons [53]. Furthermore, sulforaphane was described as neuroprotective in the Parkinson’s disease mouse model of 1-methyl-4-phenyl-1,2,3,6-tetrahydropyridine (MPTP) and 6-OHDA as it crosses the blood brain barrier [54,55]. Consistent with the aforementioned studies, the present study demonstrated the protective effect of sulforaphane against acrylamide-induced sensorimotor dysfunction and degeneration of noradrenergic axons in the murine somatosensory cortex.

First, exposure to acrylamide induced impairments in sensorimotor function as evidenced by the dose-dependent increase in hindlimb splay length (Figure S3 and Table 1). Moreover, exposure to acrylamide induced paralysis and weakness in hindlimbs as well as heightened effect of clasping when mice were suspended by the tail (Figure 1). However, these effects were abrogated by sulforaphane, including sensorimotor dysfunction (Table 1 and Figure S3). The hindlimb clasping effect has been described as a marker of neurological disorders or dysfunction, which are probably triggered by changes in neurotransmission of monoaminergic axons, including noradrenaline and serotonin within the neocortex, cerebellum and basal ganglia of mice [56]. The hindlimb clasping effect has also been considered a manifestation of motor dysfunction [57,58].

The effects of acrylamide on sensory, motor and autonomic functions have been extensively studied in laboratory animals, such as cats [59,60,61], rats [62,63,64,65], mice [66,67], monkeys [60], baboons [68,69], chickens/hens [65], and Japanese quail [70]. The results of these studies confirmed the intoxication effects of acrylamide monomer on excessive tiredness and ataxia observed in industrial workers in the 1960s [71,72]. 

Secondly, acrylamide-induced degeneration of monoaminergic axons in the primary somatosensory cortex (S1) of forelimb (S1FL), hindlimb (S1HL) and barrel field (S1BF) and secondary somatosensory cortex (S2; Figure S1), characterized by a dose-dependent and significant decrease in the density of noradrenergic axons. Notably, the extent of noradrenergic axon degeneration varied widely across the regions of the somatosensory cortex examined, with the S1BF and S2 somatosensory cortex showing the highest sensitivity (Figure 2 and Figure 5). Conversely, sulforaphane attenuated acrylamide-induced degeneration of noradrenergic axons within the primary and secondary regions of the somatosensory cortex in mice (Figure 2, Figure 3, Figure 4 and Figure 5). 

The mammalian cortex is demarcated into different regions responsible for the regulation of specific functions such as motor, sensory and cognition [73]. The somatosensory cortex forms connections with several cortical and subcortical regions of the brain [74], which allows for full representations of different parts of the body, such as face, fingers, hands, arms, feet and toes. The somatosensory cortex has, therefore, been implicated in the performance of many functions, including full representation of the body, sensory, motor, processing of painful stimuli, empathy and emotion in humans [75,76,77] and primates [78]. A converging body of evidence in the literature indicates that impairments or abnormal functioning of the somatosensory cortex contributes to deficits observed in various neurological disorders, such as aberrant sensory and motor dysfunction in humans [79,80,81,82] and monkeys [83]. At this stage, it is unclear whether the results of acrylamide-induced changes in landing foot spread as shown in the present study are directly related to severe impairments, such as degeneration of noradrenergic axons within the somatosensory cortex. Further studies are needed to investigate the exact morphological and molecular mechanisms underlying the link between somatosensory cortex and functional impairments affecting the lower limbs.

In agreement with previous studies on the pharmacological induction of the Nrf2-ARE pathway by chemical activators, the present study demonstrated that sulforaphane was associated with pronounced induction of Nrf2-antioxidant genes, evidenced by the results of mRNA expression using real-time quantitative polymerase chain reaction (RT-qPCR) in the cortex. Sulforaphane increased the nuclear translocation and expression of Nrf2 (Figure 6A) and its dependent antioxidant genes, NQO1, SOD-1, GST-M and GST-M5 additively, as well as TXNRD1 and MT-1 synergistically (Figure 6B,C,E–H). These results are consistent with those of previous studies where treatment with sulforaphane increased the expression of many Nrf2-ARE dependent antioxidants, such as NQO1, TXNRD1, HO-1 and GSTs [84,85,86]. Moreover, long-term treatment with sulforaphane in a mouse model of Parkinson’s disease inhibited rotenone-induced deficiency of locomotor activity and dopaminergic neuronal loss, together with attenuation of reactive oxygen species (ROS), MDA production as well as increased GSH levels [53].

Metallothioneins (MTs) constitute a group of cysteine-rich heavy metal binding proteins known for cellular protective functions, such as heavy metal detoxification, and protection against free radicals or oxidants among others [87,88,89,90]. MTs are considered in numerous studies to protect cells against toxicity induced by oxidants and electrophiles that can readily form reactions with sulfhydryl functional groups [91,92,93]. Electrophiles such as acrolein, acetaldehyde nitrogen mustards as well as N-ethylmaleimide readily form reactions with MTs [94]. Furthermore, Ghorbel and colleagues (2017) reported increased levels of total MT, together with increased mRNA levels of MT-I and MT-II as a major mechanism against acrylamide-induced oxidative stress in rats [95]. Several recent studies have indicated that the induction of MTs by sulforaphane is dependent on the Nrf2-ARE signaling pathway [96,97,98]. 

In the present study, the sulforaphane-induced upregulation of Nrf2-antioxidant genes known to be associated with oxidative stress was corroborated by suppression of oxidative stress, as evidenced by reduced levels of oxidative stress markers, glutathione redox ratio and MDA levels in sulforaphane-treated mice. 

Induction of the Nrf2-ARE antioxidant system by chemical activators is considered to protect against oxidative damage induced by dopamine, hydrogen peroxide and glutamine in neuronal cell lines [99,100,101]. Sulforaphane was effective in protecting against oxidative stress induced by anti-psychotic drugs in human neuroblastoma SK-N-SH cells [102]. Moreover, sulforaphane suppressed oxidative stress induced by hydrogen peroxide and paraquat, through the activation of the Nrf2-ARE pathway, thereby providing neuroprotection of rat striatal cultures [103].

However, it is noteworthy that the mRNA expression levels of HO-1 were down-regulated in sulforaphane-treated mice relative to the sulforaphane-untreated groups. Notwithstanding the fact that the induction of HO-1as a downstream gene from the Nrf2-ARE pathway has been reported in various tissues and cells to offer protection, it is becoming increasingly clear that its overexpression may induce various pathological processes, such as neurodegeneration and carcinogenesis [104]. For example, activation of HO-1 is reported to increase survival and suppression of the apoptotic pathways, and thus potentially protecting against uncontrolled proliferation, progression of cancer, metastasis and other neuronal disorders [105,106,107,108]. Moreover, it is reported that in several neurodegenerative diseases such as Alzheimer disease, Parkinson’s and multiple sclerosis, HO-1 protein is upregulated in the brain. The unexpected findings of HO-1 downregulation in sulforaphane-treated mice in the present study, therefore, signals the need for further studies to understand the related mechanisms and the net resultant impact on acrylamide-induced neurotoxicity.

Exposure of mice to acrylamide induced mRNA expression of proinflammatory cytokines, such as TNF-α and iNOS in the cortex (Figure 7A,B; Table 4; Table S3). However, sulforaphane co-treatment suppressed the acrylamide-induced upregulation of mRNA expression of proinflammatory cytokines, and thus protected against inflammation. The observed upregulation of proinflammatory cytokines suggests the potential involvement of inflammation as a major mechanism of acrylamide-induced neurotoxicity and suppression of neuroinflammation could be a mechanism mediating the neuroprotective effects of sulforaphane. There exists a considerable body of evidence in the literature indicating that sulforaphane suppressed LPS-induced cytokine secretion of TNF-α, IL-1β, IL-6, iNOS and COX-2, among others, through the activation of Nrf2, possibly by inhibition of nuclear factor kappa b (NF-kB) transcriptional activity [109,110,111,112,113]. Sulforaphane also attenuated microglia-induced inflammation in the hippocampus of LPS-exposed mice as evidenced by reduced production of pro-inflammatory cytokines, such as iNOS, IL-6 and TNF-α [36].

Exposure to acrylamide upregulated not only Nrf2-dependent transcription but also expression of Nrf2 gene itself. The mechanism for acrylamide-induced upregulation of Nrf2 gene is unknown. A previous study showed that treatment with DNA methyltransferases (Dnmts) inhibitor 5-aza-2′-deoxycytidine increased Nrf2 at both mRNA and protein levels in N2a cells through downregulation of Dnmts and DNA demethylation [114], and a recent study showed that glutathione depletion induced epigenetic alteration of vitamin D metabolism genes in the livers of high-fat diet-fed obese mice [115]. Further studies are needed to clarify how exposure to acrylamide alter the expression of Nrf2 including epigenetic mechanism of regulation on gene expression. As the major organ responsible for the metabolism of xenobiotics, the liver, is prone to chemical exposure and particularly susceptible to several toxic insults [116,117]. The present study investigated the effect of acrylamide on the liver and whether sulforaphane (which is also metabolized in the liver) protects against any acrylamide-related hepatotoxic effect. The results showed that sulforaphane provided protection against acrylamide-induced hemorrhagic necrosis of the liver (Figure S5c,d), as evidenced by clearance of necrotic lesions (Figure S5g,h).

## 4. Methods

### 4.1. Chemicals and Preparation

Acrylamide (lot #A9099, purity > 99%) and sulforaphane (lot #3200372) were purchased from Sigma Aldrich (St. Louis, MO, USA) and LKT Laboratories Inc. (St. Paul, MN), respectively. Acrylamide was freshly prepared at the beginning of each week by dissolving in a G-10 ion exchange cartridge (Organo Co., Tokyo, Japan) filtered drinking water, stored at 4 degrees Celsius and administered every day in autoclaved bottles [43]. Sulforaphane was prepared by dissolving the stock solution in normal saline just before treatment.

### 4.2. Animal Husbandry and Experimental Design

Ten-week-old male mice were used in the present study. Sixty male specific-pathogen free C57BL/6JJcl mice were purchased from CLEA Japan, Inc. (Tokyo, Japan) at 9 weeks of age and allowed to acclimatize for one week before the start of study. Mice were initially housed in separate cages of six each and had access to filtered drinking water and normal chow diet (Charles River Formula-1; CRF-1) *ad libitum*. They were housed in a controlled environment of temperature (23–25 °C), humidity (57–60%) and light (lights on at 0800 h and off at 2000 h).

**Figure 8:**
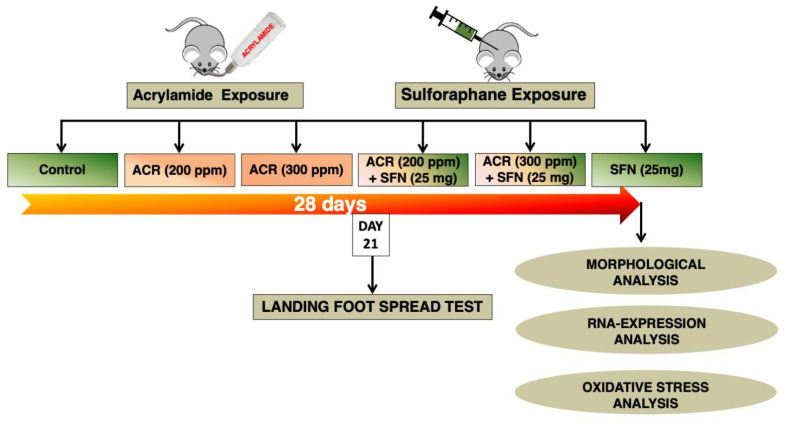
Schematic illustration of the study design. Drinking water containing acrylamide was provided daily to wild-type mice for four weeks. Each mouse also received injection of sulforaphane or saline daily for four weeks. The mice underwent functional tests (landing foot spread test) after 21 days of exposure, morphological analysis (immunostaining of neurotransmitter-specific-reactive axons; hematoxylin and eosin staining of liver), RNA-expression analysis and oxidative stress analysis after the 4-week exposure.

After the one week of acclimatization, each mouse was weighed and then assigned at random to one of six groups, each consisting of 10 mice, which were allocated into acrylamide only (0, 200 or 300 ppm) or acrylamide plus sulforaphane co-exposure groups. Groups 1 to 3 were exposed to acrylamide only at 0, 200 or 300 ppm, respectively, while groups 4, 5 and 6 were co-treated with acrylamide at 0, 200 or 300, respectively, combined with sulforaphane at 0 or 25 mg/kg body weight. Acrylamide was added to the drinking water whereas sulforaphane was administered through sub-cutaneous injections. Mice of groups 1 to 3 also received subcutaneous injections of saline (vehicle for the sulforaphane) as a measure to control any form of bias. Mice of each group (*n* = 10) for the purpose of assessment were randomly divided into groups of four and six for morphological and biochemical study respectively and treated with the compounds (acrylamide and sulforaphane) every day of the week for four weeks (Figure 8).

The protocol and experimental design of the present study were approved by the animal experiment committee of the Tokyo University of Science (Experiment approval number: Y19029 and Y20016) and strictly followed the guidelines of Tokyo University of Science on animal experiments in accordance with the Japanese act on welfare and management of animals.

### 4.3. Concentration of Acrylamide

In the present study, 300 ppm was used as the highest exposure level for acrylamide based on the findings of previous studies [67] and the fact that it matches the levels of human exposure to acrylamide at 400 ppm from a polluted drinking well water [118,119]. Moreover, in a series of preliminary studies, 300 ppm of acrylamide induced signs of neurotoxicity in experimental mice, without causing mortality. The experimental design of the present study uses oral as the route of exposure to model human exposure to acrylamide in food or water. The concentration of acrylamide (200 and 300 ppm) used in the present study are, therefore, considered relevant to human exposure as have been reported in previous studies [15,118,119,120].

### 4.4. Amount of Acrylamide Uptake in Mice

The volume of drinking water consumed together with body weight of all mice across the treatment groups were measured and recorded every day between 10:00 and 11:00 a.m. The volume of water consumption and the body weight of mice were then used to calculate the actual amount per body weight of acrylamide for each given day of exposure. Furthermore, the daily amount per body weight of acrylamide over the 28-day period of exposure was then averaged in order to obtain the mean daily amount of acrylamide per body weight. The calculated mean daily amount of acrylamide per body weight over the 28-day exposure period was 26.3 ± 1.3, and 40.4 ± 1.5 (mg/kg body weight, ± standard deviation (SD)) for the 200 and 300 ppm exposure groups respectively.

### 4.5. Hindlimb Clasping Effect

The hindlimb clasping effect was tested as described in detail previously [121,122]. Briefly, mice were suspended by their tail for 30 s and observed carefully for the extent of hindlimb clasping effect. In this test, once suspended, mice with normal neurological function extend their hindlimbs away from the abdomen with torsions of the body in an attempt to grab their tail. In contrast, mice with neurological defects retract the hindlimbs towards the abdomen, to the extent of touching both hindlimbs.

### 4.6. Landing Foot Spread Test

The landing foot spread test was performed following the protocol of the functional observatory battery testing for the effects of drugs and other chemicals on the nervous system recommended by the United States Environmental Protection Agency (USEPA) and as described previously [43]. Briefly, mice were dropped from a height of 15 cm after applying a food dye ink to the soles of the hindlimb. The distance of hindlimb spread upon landing is recorded as the hindlimb splay length. The test was carried out three times and the mean landing foot spread value was reported.

### 4.7. Tissue Harvest, Processing and Morphological Assessment

At 24 h after the last exposure of acrylamide, mice were randomly selected and euthanized for morphological and biochemical examinations.

For morphological examination, mice were selected at random (*n* = 4/group) then deeply anesthetized with intraperitoneal injection of sodium pentobarbitone (50 mg/kg). Upon confirmation of loss of sensation, the animals were transcardially perfused through the ascending aorta with 4% paraformaldehyde (4% PFA) in phosphate buffer. The perfused mice were wrapped in aluminum foil and kept on ice for a period of 1 h to increase the penetrative effect of paraformaldehyde particularly through the brain tissues. The brain was dissected out of the skull carefully and fixed for additional 24 h at 4 °C. After this, the brain was divided into three parts by cutting coronally at the anterior margin of the cerebellum and the optic chiasm and then placed in a series of 10, 20 and 30% sucrose solutions over changing intervals of 24 h each. Brain tissues were then embedded in optimal cutting temperature (OCT) medium with the use of plastic Tissue Tek cryomolds (SFJ 4566, Sakura, Japan) and then stored at –80 °C. Furthermore, liver samples were also dissected from mice and stored in 4% PFA at 4 °C until analysis.

#### Immunohistochemical Examination

The frozen OCT-embedded tissues were cryosectioned at 40 µm thickness using a cryostat (Leica CM3050S, Leica Microsystems, Wetzlar, Germany) at bregma –0.34 [123], which represents the full extent of the somatosensory cortex of mice. The frozen sections were mounted on a Matsunami MAS superfrost glass slides (Matsunami Glass Ind., Osaka, Japan) and allowed to dry at room temperature for about 1 h. Immunohistochemistry staining for noradrenaline-immunoreactive axons was performed as previously described [43]. Briefly, the air-dried sections were rinsed in Tris buffered saline (TBS; 50 mM Tris, 0.15 mM NaCl, pH 7.5–7.8) and transferred into an antigen retrieval solution containing 10 mM sodium citrate buffer (pH 8.5) that had been pre-heated to and maintained in a water bath at 80 °C for 30 min. After the incubation, the sections were cooled to room temperature together with the buffer solution and washed in Tris-buffered saline with 0.01% Tween-20 (TBST). Endogenous peroxidase activity was blocked for 20 min by incubating the sections with Bloxal, a peroxidase blocking reagent (Vector Laboratories, Burlingame, CA, USA). After triple washing in TBST, non-specific protein binding was blocked at 4 °C overnight using protein blocking reagent [1% bovine serum albumin (BSA; Sigma Aldrich), 2.5% normal horse serum (NHS; Vector Laboratories, Burlingame, CA, USA), 0.3 M glycine (Wako) and 0.1% Tween-20 (Wako)]. This was followed by brief incubation at 37 °C for 30 min followed by rinsing thrice in TBST. Endogenous interferences of avidin-biotin were blocked by incubating the sections in avidin/biotin blocking reagent (Sp-2001; Vector Laboratories), as described by the manufacturer. The sections were then incubated for 2 h at 37 °C with mouse anti-noradrenaline transporter antibody (NAT; 1:1000, #ab211463, Abcam, Japan). Following incubation with the primary antibody, the sections were washed three times and then incubated for 1 h with horse anti-mouse biotinylated secondary antibody (BA-2000; Vector Laboratories) and further washed three times in TBST. Finally, the sections were stained with the avidin-biotin peroxidase complex (Elite ABC reagent, Vector Laboratories) and visualized by reacting with diaminobenzidine peroxidase substrate as the chromogen (ImmPACT DAB (Brown) peroxidase substrate SK-4105, Vector Laboratories). After three washings in TBS Buffer, the sections were wiped off any liquid, allowed to air-dry and mounted with an aqueous mounting medium (VectaMount Mounting Medium, H-5501, Vector Laboratories).

### 4.8. Morphometric Analysis of Noradrenergic Axons

Quantification of noradrenergic axon density was conducted as described previously [43]. Uncompressed photomicrographs of the stained somatosensory cortex regions were taken with a Leica FlexCam C1 digital camera-assisted microscope (BX 50, Olympus, Tokyo). The density of noradrenergic axons was quantified in the primary somatosensory cortex (S1) of forelimb (S1FL), hindlimb (S1HL) and barrel field (S1BF) and secondary somatosensory cortex (S2) sub-regions of the somatosensory cortex (Figure S1) at Bregma –0.34 [123], using a 200 µm × 200 µm square sampling frame with the vessel analysis plugin in the ImageJ software [124]. We also determined the vascular density, which represented the ratio of the vessel area relative to the total area multiplied by 100%. These studies were conducted in 4 mice and 2 sections from each were used for analysis.

### 4.9. Tissue Processing and Histopathology

After removal, about 5 mm strips of liver tissues containing the portal section, the left lateral and medial lobes were cut and routinely processed for histopathological examination. Briefly, the trimmed liver tissue samples were dehydrated in graded ethanol concentrations (70%, 80%, 95% and 100%), cleared in two changes of xylene, impregnated and embedded in paraffin wax. Next, 5 µm thickness sections were cut using a sliding microtome (Leica) and stained with H&E. The latter were examined under a light microscope (BX100, Olympus, Tokyo) equipped with a digital camera (Leica FlexCam C1) and evaluated for hepatocellular toxicity.

### 4.10. Tissue Harvest and Biochemical Assessment

For biochemical analysis, 6 mice were randomly selected, euthanized by decapitation, and the cerebral cortex were harvested and immediately snap frozen on dry ice and stored at –80 °C until analysis. The frozen samples were crushed in an aluminum foil into a powdered form using two heavy metal rods over liquid nitrogen. The powdered tissue was carefully collected in precooled nuclease-free plastic sample tubes using a spatula. All items and utensils as mentioned were precooled in liquid nitrogen before crushing.

### 4.11. Assessment of Oxidative Stress

#### 4.11.1. Glutathione Assay (Quantification of Total and Oxidized Glutathione)

Approximately 25 mg of frozen powdered cerebral cortex tissue sample was homogenized in 250 µL of 50 mM 2-(N-morpholino) ethanesulphonic acid (MES) buffer containing 2 mM Ethylenediaminetetraacetic acid (EDTA). The homogenate was centrifuged at 10,000× *g* for 15 min at 4 °C. The supernatant was then deproteinated with an equal volume of 0.1% metaphosphoric acid (239275; Sigma Aldrich) and mixed by vortexing. The resultant mixture was allowed to stand at room temperature for 5 min and centrifuged at 2000× *g* for 2 min. The supernatant was aliquoted and stored at –20 °C until used for analysis of total and oxidized glutathione. Ninety µL of the supernatant was treated with 4.5 µL of 4 M solution of triethanolamine (TEAM; Sigma Aldrich) and immediately vortexed. For determination of total reduced glutathione (GSH), 30 µL TEAM-treated sample was diluted 10-fold using the MES buffer. Subsequently, 50 µL of the diluted sample was added to 150 µL freshly prepared assay cocktail (MES buffer, reconstituted cofactor mixture, reconstituted enzyme mixture, distilled water and reconstituted DNTB) and incubated for 25 min, following which the mixture was assayed at 405 nm with a microplate reader (Gen5™ Secure, BioTek^®^ Instruments, Inc.)

For determination of oxidized glutathione (GSSG), 30 µL of the TEAM-treated sample was diluted 5-fold with MES buffer and 200 µL of this diluted solution was derivatized with 2 µL of 2-vinylpyridine (Sigma Aldrich). The mixture was vortexed and incubated for 1 h at room temperature. Fifty µL of the derivatized sample was then mixed with 150 µL of freshly prepared assay cocktail as explained earlier, incubated for 25 min in the dark and then assayed at 405 nm on a microplate reader. The concentrations of GSH and GSSG were calculated using absorbance in an equation of the line obtained from the GSSG standard curve provided in the GSH assay kit (#703002, Cayman Chemical Company, Ann Arbor, MI) and expressed as micro-moles of GHS or GSSG per mg protein.

#### 4.11.2. Thiobarbituric Acid Reactive Substances (TBARS) Assay

MDA was measured using TBARS assay (#10009055, Cayman Chemical Company) following the instructions provided by the manufacturer. Briefly 25 mg of powdered cerebral cortex tissue was homogenized in 250 µL of radioimmunoprecipitation assay buffer (RIPA) containing protease inhibitors on ice and then centrifuged for 10 min at 1600× *g* and 4 ˚C. The supernatant was then aliquoted and kept on ice. A mixture containing 100 µL of the sample supernatant together with 100 µL of sodium dodecyl sulfate (SDS) solution and 4 mL of color reagent was prepared in 5-mL vial and boiled vigorously for one hour. This was to enable the reaction between the sample and thiobarbituric acid (TBA). Next, the vial was removed from boiling water and placed in ice bath for 10 min to stop the reaction. The vial was then centrifuged for 10 min at 1600× *g* and 4 °C. Subsequently, 150 µL was loaded in duplicates into microplate and read at 532 nm with a microplate reader (Gen5™ Secure, BioTek^®^ Instruments, Inc.). MDA concentration was calculated using the MDA colorimetric standard curve and expressed as micromoles of MDA per mg protein.

### 4.12. Total mRNA Isolation, cDNA Synthesis and Real-Time Quantitative Polymerase Chain Reaction (PCR)

Total messenger RNA (mRNA) was isolated from the cerebral cortex using the RNeasy Lipid Tissue Mini Kit (Qiagen Benelux B.V., Venlo, Netherlands) and according to the instructions provided by the manufacturer. The concentration of the extracted mRNA following elution with RNase-free water was measured using a NanoDrop 2000 spectrophotometer (Thermo Fisher Scientific, Waltham, MA, USA). The quality of mRNA was determined by confirming that the A260/A280 ratio was ≥2.0 after measuring absorbance at 260 nm and 280 nm. Complementary DNA (cDNA) was then synthesized by using 4µg of total extracted RNA with SuperScript III reverse transcriptase (Invitrogen, Carlsbad, CA, USA) according to the instructions supplied by the manufacturer. Real-time quantitative PCR was performed by using the THUNDERBIRD^®^ SYBR^®^ qPCR Mix (Toyobo Co., Osaka) and the AriaMx Real-Time PCR System (Agilent Technologies, Inc., Santa Clara, CA). A three-step real-time PCR amplification reaction comprising an initial denaturation step at 95 °C for 1 min, followed by an amplification step of 45 denaturation cycles at 95 °C for 15 s, primer annealing at 60 °C for 30 s, extension at 72 °C for 60 s and reading of plate was utilized. A melting curve step from 55 to 95 °C with 0.5 °C increments for 5 s followed by a plate read was also incorporated. A standard curve constructed using serial concentrations of diluted cDNA samples from the control group was used to quantify the relative expression level of each gene. The latter was calculated by standardization to the endogenous mRNA levels of the housekeeping gene β-actin. The mRNA expression levels of Nrf2-antioxidant genes: Nrf2, heme oxygenase 1 (HO-1), NAD(P)H:quinone oxidoreductase 1 (NQO1), superoxide dismutase 1 (SOD 1), glutathione-s-transferase mu (GST-M), glutathione-s-transferase mu-5 (GST-M5), thioredoxin reductase 1 (Txnrd1) and metallothionein 1 (MT-1), as well as genes of several pro-inflammatory cytokines, including tumor necrosis factor alpha (TNF-α), inducible nitric oxide synthase (iNOS), interleukin 1 beta (IL-1β), IL-6 and cyclooxygenase 2 (COX-2) were analyzed. Primer sequences for the various genes are listed in Table 6.

### 4.13. Statistical Analysis

Statistical analysis was performed using GraphPad Prism version 8.0 (GraphPad Software, La Jolla, CA, USA) or JMP (version 14, SAS Institute, Cary, NC, USA). Data are expressed as mean ± standard deviation (SD) or ±standard error of the mean (SEM), as indicated. Differences among groups were analyzed by one-way ANOVA followed by Dunnett’s multiple comparison test. Single regression analysis was carried out on the dose of acrylamide in each mouse treatment groups (acrylamide only and acrylamide plus sulforaphane group) to determine the effects of sulforaphane and trend with the dose of acrylamide by the use of dummy variables for treatment. Multiple regression analysis with dummy variable (0: without sulforaphane and 1: with sulforaphane) was carried out to determine the interaction between the dose of acrylamide and sulforaphane treatment. A model of multiple regression analysis without interaction was used to test the effects of the dose of acrylamide and sulforaphane when their interaction was not significant. A probability (p) of <0.05 denoted the presence of a statistically significant difference.

## 5. Conclusions

We have confirmed in the present study that in mice, acrylamide is neurotoxic, causing hindlimb dysfunction, degeneration of monoaminergic axons, as well as hepatotoxic, and that activation of the Nrf2 signaling pathway through co-administration of sulforaphane abrogates the ill-effects of acrylamide. The sulforaphane-mediated induction of the Nrf2 signaling pathway resulted in upregulation of Nrf2 and its downstream genes, leading to protection against acrylamide-induced neurotoxicity, through prevention of oxidative stress together with suppression of pro-inflammatory cytokine gene upregulation. The present study shows that activation of the Nrf2 signaling pathway through dietary phytochemicals such as sulforaphane attenuates acrylamide-induced neuro-hepatotoxicity and further provides a scientific basis for nutrient recommendations in the preventive modulation of electrophile-induced diseases.

## Figures and Tables

**Table 1:** Changes in body weight and landing foot spread according to the dose of acrylamide and sulforaphane treatment.

Test Parameters	Treatment	Concentration of Acrylamide (ppm)			Simple Regression	Multiple Regression (*p* Value)		
		0	200	300	Regression Coefficient of ACR (*p* Value)	Interaction of ACR and SFN	Regression Coefficient of ACR	Regression Coefficient of SFN
Body Weight (g)	*SFN* (−)	25.4 ± 1.2	24.8 ± 1.1	23.3 ± 1.5 *	−0.007 (0.002) g/ppm	0.004 (0.17)	−0.007 (0.0005) g/ppm	0.25 (0.42)/mg
	*SFN* (+)	25.1 ± 1.3	24.9 ± 0.8	24.1 ± 1.1	−0.003 (0.07) g/ppm			
Landing foot spread (cm)	*SFN* (−)	2.7 ± 0.4	3.7 ± 0.5 *	4.5 ± 0.7 *	0.006 (<0.0001) cm/ppm	−0.002 (0.12)	0.006 (<0.0001) cm/ppm	−0.37 (0.02)/mg
	*SFN* (+)	2.5±0.5	3.6±0.3 *	3.7±1.0 *	0.004 (0.0003) cm/ppm			

Abbreviation: ACR, acrylamide; SFN, sulforaphane. Data are mean ± standard deviation (SD). * *p* < 0.05, compared with the corresponding treatment control (by ANOVA followed by Dunnett’s multiple comparison) for body weight and landing foot spread test (*n* = 10). Mice were exposed daily to acrylamide in drinking water and co-administered with sulforaphane in saline for 28 days. Simple regression analysis in each treatment (*n* = 30 per each treatment for body weight and landing foot) and tests for interaction in multiple regression model (*n* = 60 for landing foot spread and body weight) with dummy variables (0: acrylamide only and 1: sulforaphane treatment) for treatment were conducted for body weight and landing foot spread. Since interaction was not significant for body weight and landing foot spread, multiple regression analysis in a model without interaction (*n* = 60) was conducted to estimate the effect of acrylamide or sulforaphane co-treatment.

**Table 2:** Results of regression analysis of the effects of acrylamide and sulforaphane co-treatment on the density of noradrenergic axons in primary and secondary somatosensory cortex.

Test Parameter	Region	Treatment	Simple Regression	Multiple Regression (*p* Value)		
			Regression Coefficient of ACR (*p* Value)	Interaction of ACR and SFN	Regression Coefficient of ACR	Regression Coefficient of SFN
Density of noradrenergic axons (%)	S1HL	*SFN* (−)	−0.03 (0.02) %/ppm	0.02 (0.07)	−0.03 (0.001) %/ppm	1.4 (0.3)/mg
		*SFN* (+)	−0.008 (0.03) %/ppm			
	S1BF	*SFN* (−)	−0.04 (0.001) %/ppm	−0.03 (0.009)	-	-
		*SFN* (+)	−0.007 (0.24) %/ppm			
	S1FL	*SFN* (−)	−0.02 (0.01) %/ppm	0.01 (0.23)	−0.02 (0.001) %/ppm	0.41 (0.70)/mg
		*SFN* (+)	−0.01 (0.02) %/ppm			
	S2	*SFN* (−)	−0.04 (<0.0001) %/ppm	0.03 (0.002)	-	-
		*SFN* (+)	−0.007 (0.29) %/ppm			

Abbreviation: ACR, acrylamide; SFN, sulforaphane. Primary somatosensory cortices (S1BF: barrel field; S1FL: forelimb; S1HL: hindlimb) and secondary somatosensory cortex (S2). Simple regression analysis for each treatment (*n* = 12 per treatment) and test for interaction in multiple regression model (*n* = 24 per treatment group) with dummy variables (0: wild type and 1: sulforaphane treated mice) for treatment were conducted for noradrenergic axons. When interaction was not significant for density of noradrenergic axons in S1HL and S1FL, multiple regression analysis in a model without interaction (*n* = 24) was conducted to estimate the effect of acrylamide or sulforaphane treatment. Since significant interaction was found in density of noradrenergic axons in S1BF and S2, the effect of acrylamide or sulforaphane treatment was not tested in multiple regression analysis.

**Table 3:** Results of regression analysis of the effects of acrylamide and sulforaphane on mRNA expression of Nrf2-antioxidants in the cerebral cortex.

Test Parameters	Treatment	Simple Regression	Multiple Regression (*p* value)		
		Regression Coefficient of ACR (*p* Value)	Interaction of ACR and SFN	Regression Coefficient of ACR	Regression Coefficient of SFN
SOD-1	*SFN* (−)	0.0005 (0.02)/ppm	0.0007 (0.09)	0.0005 (0.10)/ppm	0.26 (<0.0001)/mg
	*SFN* (+)	0.001 (0.005)/ppm			
NQO1	*SFN* (−)	0.002 (0.04)/ppm	0.00008 (0.95)	0.002 (0.02)/ppm	0.33 (0.05)/mg
	*SFN* (+)	0.002 (0.02)/ppm			
HO-1	*SFN* (−)	0.002 (0.006)/ppm	−0.002 (0.06)	0.002 (0.002)/ppm	-0.26 (0.02)/mg
	*SFN* (+)	0.0004 (0.45)/ppm			
GST-M5	*SFN* (−)	0.0006 (0.08)/ppm	0.0003 (0.45)	0.0006 (0.07)/ppm	0.23 (0.0001)/mg
	*SFN* (+)	0.0009 (0.01)/ppm			
GST-M	*SFN* (−)	0.001 (0.004)/ppm	0.0003 (0.67)	0.001 (0.003)/ppm	0.26 (0.002)/mg
	*SFN* (+)	0.002 (0.002)/ppm			
NRF2	*SFN* (−)	0.0005 (0.02)/ppm	0.0003 (0.33)	0.0005 (0.05)/ppm	0.07 (0.11)/mg
	*SFN* (+)	0.0008 (0.01)/ppm			
TXNRD1	*SFN* (−)	−0.0001 (0.81)/ppm	0.002 (0.004)	-	-
	*SFN* (+)	0.002 (<0.0001)/ppm			
MT-1	*SFN* (−)	0.003 (0.0008)/ppm	0.002 (0.03)	-	-
	*SFN* (+)	0.005 (<0.0001)/ppm			

Abbreviation: ACR, acrylamide; SFN, sulforaphane. Simple regression analysis for each genotype (*n* = 18 per each treatment) and test for interaction in multiple regression model (*n* = 36 per treatment group) with dummy variable (0: acrylamide only and 1: sulforaphane co-treated mice) for treatment were conducted for SOD-1, NQO1, HO-1, GST-M5, GST-M, Nrf2, Txnrd1 and MT-1. Since interaction was not significant for SOD-1, NQO1, HO-1, GST-M5, GST-M and Nrf2, multiple regression analysis in a model without interaction (*n* = 36) was conducted to estimate the effect of acrylamide and sulforaphane. As significant interaction was found for Txnrd1 and MT-1, the effect of acrylamide and sulforaphane was not tested in multiple regression.

**Table 4:** Results of regression analysis for the effects of acrylamide and sulforaphane on mRNA expression of pro-inflammatory cytokines in the cerebral cortex.

Test Parameters	Treatment	Simple Regression	Multiple Regression (*p* Value)		
		Regression Coefficient of ACR (*p* Value)	Interaction of ACR and SFN	Regression Coefficient of ACR	Regression Coefficient of SFN
TNF-α	*SFN* (−)	0.003 (0.01)/ppm	−0.002 (0.09)	0.003 (0.002)/ppm	−0.03 (0.80)/mg
	*SFN* (+)	0.0007 (0.22)/ppm			
iNOS	*SFN* (−)	0.002 (0.008)/ppm	−0.001 (0.21)	0.002 (0.001)/ppm	−0.21 (0.07)/mg
	*SFN* (+)	0.001 (0.05)/ppm			
IL-Iβ	*SFN* (−)	0.0002 (0.45)/ppm	−0.00009 (0.86)	0.0002 (0.56)/ppm	−0.13 (0.05)/mg
	*SFN* (+)	0.0001 (0.78)/ppm			
IL-6	*SFN* (−)	0.0004 (0.36)/ppm	−0.0002 (0.70)	0.0004 (0.27)/ppm	−0.22 (0.003)/mg
	*SFN* (+)	0.0002 (0.47)/ppm			
COX-2	*SFN* (−)	−0.00001 (0.99)/ppm	−0.0003 (0.74)	−0.00001 (0.98)/ppm	0.11 (0.27)/mg
	*SFN* (+)	−0.0003 (0.60)/ppm			

Abbreviation: ACR, acrylamide; SFN, sulforaphane. Simple regression analysis for each genotype (*n* = 18 per each treatment) and test for interaction in multiple regression model (*n* = 36 per treatment group) with dummy variables (0: acrylamide only and 1: sulforaphane co-treated mice) for treatment were conducted for TNF-α, iNOS, IL-1β, IL-6 and COX-2. Since interaction was not significant for TNF-α, iNOS, IL-1β, IL-6 and COX-2, multiple regression analysis in a model without interaction (*n* = 36) was conducted to estimate the effects of acrylamide and sulforaphane.

**Table 5:** Effects of different doses of acrylamide and sulforaphane co-treatment on the expression levels of oxidative stress markers in cerebral cortex.

Test Parameters	Treatment	Acrylamide Concentration (ppm)			Simple Regression	Multiple Regression (*p* Value)		
		0	200	300	ACR Regression Coefficient (*p* Value)	Interaction of ACR and SFN	ACR Regression Coefficient	SFN Regression Coefficient
Total Glutathione (GSH + GSSG, µM)	*SFN* (−)	92.1 ± 12.3	94.4 ± 18.0	103.3 ± 21.3	0.034 (0.32)/ppm	−0.05 (0.29)	0.03 (0.28)/ppm	16.69 (0.004)/mg
	*SFN* (+)	115.7 ± 16.1	111.8 ± 6.7	112.2 ± 20.3	−0.013 (0.66)/ppm			
Glutathione Disulfide (GSSG, µM)	*SFN* (−)	2.7 ± 2.8	2.8 ± 2.2	3.7 ± 1.5	0.003 (0.46)/ppm	−0.004 (0.51)	0.003 (0.45)/ppm	−0.36 (0.62)/mg
	*SFN* (+)	2.5 ± 1.4	3.6 ± 2.3	2.0 ± 2.4	−0.001 (0.86)/ppm			
GSSG/GSH ratio (×10^−2^)	*SFN* (−)	3.3 ± 3.7	3.2 ± 2.9	4.0 ± 2.4	0.002 (0.73)/ppm	−0.002 (0.73)	0.002 (0.67)/ppm	−1.11 (0.19)/mg
	*SFN* (+)	2.1 ± 1.1	3.2 ± 2.0	1.8 ± 1.9	−0.0003 (0.92)/ppm			
MDA (µM)	*SFN* (−)	6.5 ± 2.2	6.6 ± 2.2	8.7 ± 1.1	0.006 (0.10)/ppm	−0.015 (0.003)	-	-
	*SFN* (+)	8.5 ± 1.6	7.0 ± 1.3	5.9 ± 1.6 *	−0.008 (0.008)/ppm			

Abbreviation: ACR, acrylamide; GSH, glutathione; GSSG/GSH ratio, glutathione redox ratio; MDA, malondialdehyde; SFN, sulforaphane. Data are mean ±SD. * *p* < 0.05, compared to the corresponding treatment control (by ANOVA followed by Dunnett’s multiple comparison). (*n* = 6). Simple regression analysis for each genotype (*n* = 18 per each treatment) and test for interaction in multiple regression model (*n* = 36 per treatment group) with dummy variables (0: ACR only and 1: sulforaphane co-treated mice) for treatment were conducted for total glutathione, glutathione disulfide, GSSG/GSH ratio and MDA. Since interaction was insignificant for total glutathione, glutathione disulfide and GSSG/GSH ratio, multiple regression analysis was conducted in a model without interaction (*n* = 36) to estimate the effect of acrylamide or sulforaphane co-treatment. Since significant interaction was found for MDA, the effect of acrylamide or sulforaphane was not tested in multiple regression model.

**Table 6:** Gene primers used for real-time quantitative polymerase chain reaction (qRT-PCR).

Gene	PRIMER SEQUENCES	REFERENCES
Nfe2l2 (Nrf2)	F: CGAGATATACGCAGGAGAGGTAAGAR: GCTCGACAATGTTCTCCAGCTT	[125]
Keap-1	F: GATCGGCTGCACTGAACTGR: GGCAGTGTGACAGGTTGAAG	[126]
Gst-M (GSTµ)	F: CTGAAGGTGGAATACTTGGAGCR: GCCCAGGAACTGTGAGAAGA	[127]
GST-M5	F: AGAAACGGTACATCTGTGGGGR: GGATGGCGTTACTCTGGGTG	[128]
HO-1	F: CACAGATGGCGTCACTTCCGTCR: GTGAGGACCCACTGGGAGGAG	[129]
NQO1	F: AGCGTTCGGTATTACGATCCR: AGTACAATCAGGGCTCTTCTCG	[130]
SOD-1	F: CAGGACCTCATTTTAATCCTCACR: TGCCCAGGTCTCCAACAT	
TXNDR1	F: GGGTCCTATGACTTCGACCTGR: AGTCGGTGTGACAAAATCCAAG	[131]
MT-1	F: ACCTCCTGCAAGAAGAGCTGR: GCTGGGTTGGTCCGATACTA	[132]
TNF-α	F: CATCTTCTCAAAATTCGAGTGACAAR: TGGGAGTAGACAAGGTACAACCC	[36]
iNOS	F: CCTCCTTTGCCTCTCACTCTTR: AGTATTAGACGCGTGGCATGG	[133]
IL-1β	F: CTGGTGTGTGACGTTCCCATTAR: CCGACAGCACGAGGCTTT	
IL-6	F: CCTACCCCAATTTCCAATGCTR: TATTTTCTGACCACAGTGAGGAAT	
COX-2	F: TTCGGGAGCACAACAGAGTR: TAACCGCTCAGGTGTTGCAC	[36]
β-ACTIN	F: TCCTTCCTGGGCATGGAGR: AGGAGGAGCAATGATCTTGATCTT	[133]

## Supplementary Materials

They are available online at https://www.mdpi.com/article/10.3390/ijms22115995/s1.

## Abbreviations

ACR: Acrylamide; ANOVA, Analysis of Variance; ARE, Antioxidant response element; b.w., Body weight; bZIP, Basic-region leucine zipper; CNC, Cap ‘n’ collar; COX-2, Cyclooxygenase 2; EpRE, Electrophile response element; GSH, Glutathione; GSSG, Glutathione disulfide; Gst, Glutathione S-transferase; GST-M, Glutathione S transferase mu; GST-M5, Glutathione S transferase mu5; HO-1, Heme oxygenase; IL, Interleukin; IL-1β, Interleukin 1beta; IL-6, Interleukin 6; iNOS, Inducible nitric oxide synthase; KEAP1, Kelch-like ECH-associated protein 1; MDA, Malondialdehyde; MES, 2-(N-morpholino)ethane sulfonic acid; MT-1, Metallothionein 1; Neh, Nrf2-ECH homology; NA, Noradrenaline; NAT, Noradrenaline transporter; NQO1, NAD(P)H:quinone oxidoreductase 1; Nrf2, Nuclear factor E2-related factor 2; RIPA buffer, Radioimmunoprecipitation assay buffer; ROS, Reactive oxygen species; S1BF, primary somatosensory cortex, barrel field; S1HL, primary somatosensory cortex hindlimb; S1FL, primary somatosensory cortex forelimb; S2, secondary somatosensory cortex; sMaf, small musculoaponeurotic fibrosarcoma; SOD-1, Superoxide dismutase 1; SFN, Sulforaphane; TBARS, Thiobarbituric acid reactive substances; TEAM, triethanolamine; TNF-α, Tumor necrosis factor-alpha.
