# Supplementary material for: Nrf2 Activation Attenuates Acrylamide-Induced Neuropathy in Mice
Source: Int J Mol Sci. 2021 Jun 1;22(11):5995. doi: 10.3390/ijms22115995 (PMC8199319; doi:10.3390/ijms22115995)
Supplement: Supplementary file 1 [file ijms-22-05995-s001.zip › ijms-1246267-supplementary.pdf]

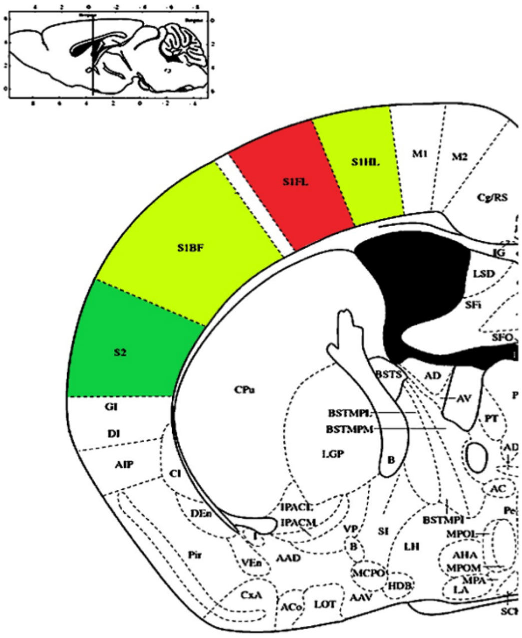

Figure S1. The mouse brain in stereotaxic coordinates showing the regions of the somatosensory cortex [124]. Immunohistochemistry for noradrenergic axons (noradrenaline transporter; NAT) was performed and quantified within the primary (S1BF, S1HL, S1FL) and secondary (S2) somatosensory cortex.

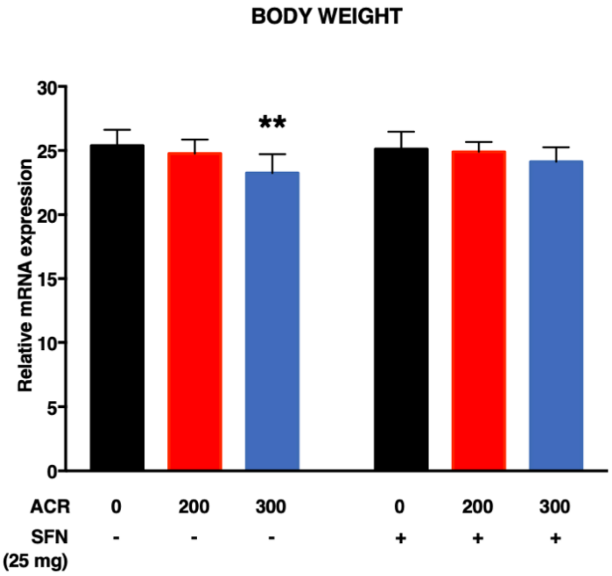

Figure S2. Effects of 4-week acrylamide and sulforaphane administration on body weight. Data are mean ± SD. *P<0.05, **P<0.001compared to the corresponding treatment control (by ANOVA followed by Dunnett's multiple comparison). [n=10].

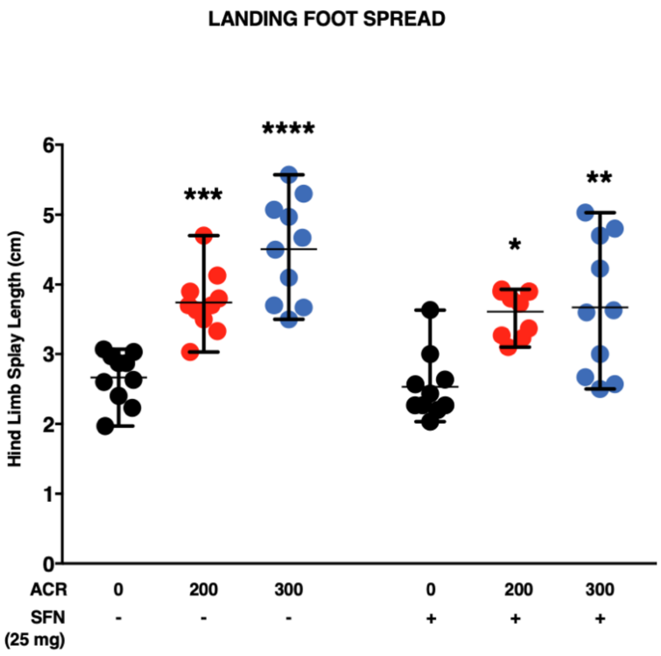

Figure S3. Effects of acrylamide on landing foot spread in mice. Acrylamide increased hindlimb splay in mice and sulforaphane abrogated this effect. Data are mean ±SD. *P<0.05, ** $p < 0.01$, *** $p < 0.001$, **** $p < 0.0001$, compared to the corresponding treatment control (by ANOVA followed by Dunnett's multiple comparison). (n=10).

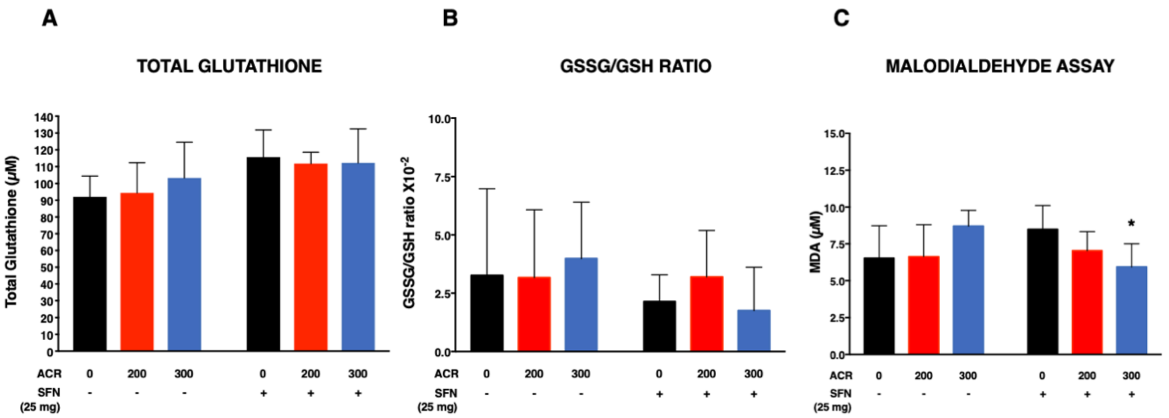

Figure 4. Effects of 4-week exposure to acrylamide and sulforaphane treatment on induction of markers of oxidative stress in the cerebral cortex; total glutathione, glutathione redox ratio (GSSG/GSH) and malondialdehyde (MDA). Data are mean ± SD. *P < 0.05, compared to the corresponding treatment control (by ANOVA followed by Dunnett's multiple comparison). [n=6]

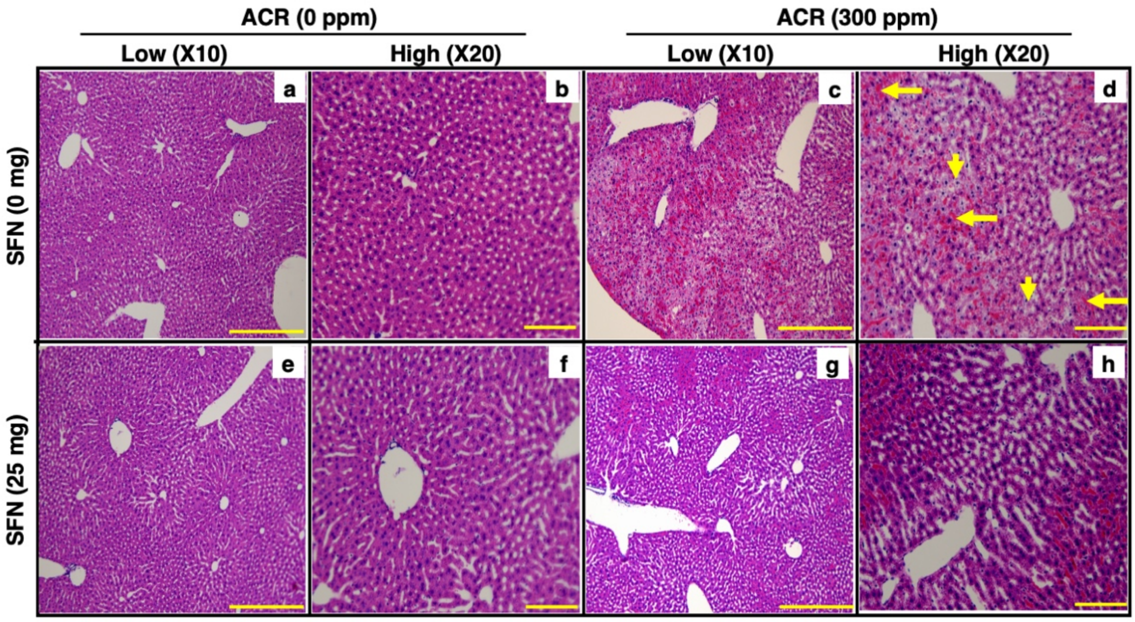

Figure S5. Representative photomicrographs of H&E-stained histological sections of the liver following exposure to acrylamide and sulforaphane for 4 weeks. Acrylamide induced extensive necrosis (arrowheads) and severe hemorrhage (arrows). Photomicrographs of the liver of acrylamide-exposed mice (a-d), and acrylamide plus sulforaphane-treated mice (e-h). Note the limited hemorrhage and clearance of necrotic lesions in sulforaphane-treated mice. Scale bars= 250 μm (a, c, e and g) and 40 μm (b, d, f and h).

Table S1. Effects of different doses of acrylamide and sulforaphane treatment on the density of noradrenergic axons in primary somatosensory cortex (S1HL, S1BF and S1FL) and secondary somatosensory cortex (S2)

Region	Treatment	Concentration of acrylamide (ppm)		
		0	200	300
S1HL	*SFN (-)*	29.3±2.4	23.6±5.4	20.7±5.4*
	SFN (+)	27.3±1.4	25.5±1.6	25.0±1.1
S1BF	*SFN (-)*	29.3±5.6	19.2±1.6*	18.7±1.8*
	SFN (+)	26.4±1.0	22.3±1.9*	24.9±27.0
S1FL	*SFN (-)*	29.2±2.6	23.2±3.9	22.6±2.9*
	SFN (+)	27.7±1.1	24.4±3.0	24.1±1.0*
S2	*SFN (-)*	28.5±2.4	19.8±1.8*	18.1±2.6*
	SFN (+)	26.2±2.4	26.1±3.9	23.9±0.5

Data are mean ±SD.
*p<0.05, compared with the corresponding treatment control by Dunnett's multiple comparison following ANOVA for the density of noradrenergic axons in S1HL, S1BF, S1FL and S2 (n=4).
Primary somatosensory cortices (S1BF: barrel field; S1FL: forelimb; S1HL: hindlimb), secondary somatosensory cortex (S2).

Table S2. Effects of different doses of acrylamide and sulforaphane treatment on the relative mRNA expression levels of Nrf2-downstream antioxidant genes in the cerebral cortex

Test Parameter	Treatment	Concentration of acrylamide (ppm)		
		0	200	300
SOD-1	*SFN (-)*	1.0±0.1	1.1±0.1	1.2±0.1*
	SFN (+)	1.2±0.1	1.3±0.1	1.6±0.3*
NQO1	*SFN (-)*	1.0±0.1	1.2±0.1	1.7±0.9
	SFN (+)	1.3±0.4	1.5±0.3	2.1±0.6*
HO-1	*SFN (-)*	1.0±0.2	1.4±0.3	1.7±0.5*
	SFN (+)	1.0±0.2	1.1±0.4	1.1±0.3
GST-M5	*SFN (-)*	1.0±0.1	1.1±0.2	1.2±0.2
	SFN (+)	1.2±0.1	1.2±0.1	1.5±0.2*
GST-M	*SFN (-)*	1.0±0.1	1.3±0.2	1.4±0.3*
	SFN (+)	1.2±0.1	1.4±0.1	1.7±0.4*
NRF2	*SFN (-)*	1.0±0.1	1.1±0.1	1.2±0.1*
	SFN (+)	1.0±0.1	1.1±0.1	1.3±0.2*
TXNRD1	*SFN (-)*	1.0±0.4	0.9±0.2	1.0±0.2
	SFN (+)	0.6±0.1	1.0±0.3*	1.2±0.2*
MT-1	*SFN (-)*	1.0±0.2	1.5±0.3	1.9±0.6*
	SFN (+)	0.8±0.2	1.6±0.6*	2.5±0.3*

Data are mean ±SD.
*p<0.05, compared to the corresponding genotype control by Dunnett's multiple comparison following ANOVA (n=6).

Table S3. Effects of different doses of acrylamide and sulforaphane treatment on the relative mRNA expression levels of pro-inflammatory cytokines in the cerebral cortex

Test Parameter	Treatment	Concentration of acrylamide (ppm)		
		0	200	300
TNF-α	*SFN (-)*	1.0±0.3	1.2±0.2	1.8±0.7*
	SFN (+)	1.1±0.3	1.4±0.3	1.3±0.3
iNOS	*SFN (-)*	1.0±0.5	1.3±0.3	1.7±0.3*
	SFN (+)	0.9±0.3	1.2±0.2	1.2±0.3
IL-Iβ	*SFN (-)*	1.0±0.1	1.1±0.1	1.1±0.2
	SFN (+)	0.9±0.2	1.0±0.3	0.9±0.2
IL-6	*SFN (-)*	1.0±0.2	1.2±0.3	1.1±0.2
	SFN (+)	0.8±0.1	1.0±0.2	0.9±0.1
COX-2	*SFN (-)*	1.0±0.3	1.1±0.3	1.0±0.3
	SFN (+)	1.2±0.2	1.1±0.3	1.1±0.3

Data are mean ±SD.
*p<0.05, compared to the corresponding genotype control by Dunnett's multiple comparison following ANOVA (n=8).
